# Roadmap and Considerations for Genome Editing in a Non-Model Organism: Genetic Variations and Off-Target Profiling

**DOI:** 10.3390/ijms252312530

**Published:** 2024-11-22

**Authors:** Hanin Wattad, Jonathan Molcho, Rivka Manor, Simy Weil, Eliahu D. Aflalo, Vered Chalifa-Caspi, Amir Sagi

**Affiliations:** 1 Department of Life Sciences, Ben-Gurion University of the Negev, P.O. Box 653, Beer-Sheva 8410501, Israel; haninw@post.bgu.ac.il (H.W.); molchoj@post.bgu.ac.il (J.M.); mnor@bgu.ac.il (R.M.); simmi@bgu.ac.il (S.W.); aflaloe@bgu.ac.il (E.D.A.); 2 The National Institute for Biotechnology in the Negev, Ben-Gurion University of the Negev, P.O. Box 653, Beer-Sheva 8410501, Israel; 3 Department of Life Sciences, Achva Academic College, Arugot 7980400, Israel; 4 Bioinformatics Core Facility, Ilse Katz Institute for Nanoscale Science & Technology, Ben-Gurion University of the Negev, Beer-Sheva 8410501, Israel; veredcc@bgu.ac.il

**Keywords:** CRISPR/Cas, GUIDE-seq, *Macrobrachium rosenbergii*, sex chromosomes, single nucleotide polymorphisms (SNPs), repetitive sequences

## Abstract

The CRISPR/Cas genome editing approach in non-model organisms poses challenges that remain to be resolved. Here, we demonstrated a generalized roadmap for a de novo genome annotation approach applied to the non-model organism *Macrobrachium rosenbergii*. We also addressed the typical genome editing challenges arising from genetic variations, such as a high frequency of single nucleotide polymorphisms, differences in sex chromosomes, and repetitive sequences that can lead to off-target events. For the genome editing of *M. rosenbergii*, our laboratory recently adapted the CRISPR/Cas genome editing approach to embryos and the embryonic primary cell culture. In this continuation study, an annotation pipeline was trained to predict the gene models by leveraging the available genomic, transcriptomic, and proteomic data, and enabling accurate gene prediction and guide design for knock-outs. A next-generation sequencing analysis demonstrated a high frequency of genetic variations in genes on both autosomal and sex chromosomes, which have been shown to affect the accuracy of editing analyses. To enable future applications based on the CRISPR/Cas tool in non-model organisms, we also verified the reliability of editing efficiency and tracked off-target frequencies. Despite the lack of comprehensive information on non-model organisms, this study provides an example of the feasibility of selecting and editing specific genes with a high degree of certainty.

## 1. Introduction

Genome editing utilizing the clustered regularly interspaced palindromic repeats (CRISPR)/CRISPR-associated protein (Cas9) system has evolved into a powerful tool for modifying the genomes of both model and non-model organisms. In model species, such as fruit flies (*Drosophila*) [1], zebrafish (*Danio rerio*) [2,3], mice (*Mus musculus*) [4] and the nematode *Caenorhabditis elegans* [5], CRISPR/Cas has enabled highly efficient genome editing, allowing gene knock-outs, gene knock-ins, and precise point mutations. In contrast, for non-model organisms, there is often a lack of comprehensive genetic and genomic databases for identifying and analyzing genes, pathways, and regulatory elements. This lacuna makes the utilization of precise CRISPR/Cas genome editing in non-model organisms more challenging than that in well-studied model organisms. Thus, there is a necessity to develop a genome editing approach for non-model organisms as a crucial tool for studying diverse biological systems. By expanding the range of organisms that can be easily manipulated for future genetic studies, novel and practical applications can be developed in the field of genetically edited organisms.

CRISPR/Cas genome editing has shown promising potential for applications in crustacean and fish aquacultures and for non-model aquatic organisms, even though genome editing for these organisms is still in its infancy compared to that for other taxa [6]. Nonetheless, there have been some preliminary breakthroughs in crustacean species, such as *Daphnia magna* [7], *Exopalaemon carinicauda* [8], *Parhyale hawaiensis* [9], and *Macrobrachium nipponense* [10]. Of particular interest is the gene-editing platform facilitated by CRISPR/Cas for the giant freshwater prawn *M. rosenbergii* that was established in our laboratory through the use of microinjection in embryos and nucleofection in an embryonic primary cell culture [11].

*M. rosenbergii* is a commercially important aquaculture species [12] that has been relatively well studied, as is evidenced by the publication of as many as 215 research articles since 2022, according to the Web of Science. Nonetheless, as it is a non-model organism, there is a lack of extensive databases for *M. rosenbergii* compared to model organisms. Thus, applying CRISPR/Cas to *M. rosenbergii* presents unique challenges, such as those posed by genetic variations and off-target events. Nevertheless, the successful use of CRISPR/Cas in *M. rosenbergii* [11] is offering possibilities for the genetic improvement that will make a significant contribution to aquaculture production (e.g., for enhancing desirable traits and manipulating sex determination).

Successful gene editing hinges on the crucial first step of high-quality genome annotation [13,14,15]. For non-model organisms, complete and accurate genome sequences might not be available, and, hence, comprehensive and detailed gene annotations remain incomplete or unreliable, especially for coding regions, thereby posing a significant challenge in gene editing. Specifically, single nucleotide polymorphisms (SNPs) and repetitive sequences in autosomal chromosomes may pose challenges for CRISPR/Cas genome editing in non-model organisms, particularly regarding specificity, efficiency, and the ability to modify target genomic regions [16,17,18].

To understand the complexity of these challenges, let us take a closer look at the CRISPR/Cas technology. This technology has been used to eliminate entire chromosomes, including sex chromosomes, such as the Y chromosome in mice [19]. It is noteworthy that the sex chromosomes (X/Y or Z/W) in both model and non-model species often have unique repetitive sequences and genetic polymorphisms that play essential roles in the evolution of sex determination systems and potential speciation [20,21,22]. Genetic variations, such as SNPs and repetitive sequences, and the inherent differences between sex chromosomes, can also contribute to off-target effects in CRISPR/Cas genome editing. In particular, the CRISPR/Cas system can potentially induce unintended double-strand breaks at genomic sites that are similar, but not identical, to on-target sequences, leading to off-target manipulations. This can result in unintended mutations and structural variations in the genome, which, in some cases, may have serious consequences by disrupting the function or regulation of non-targeted genes. Since the prevention of off-target effects is critical for the safe and effective use of CRISPR/Cas genome-editing technology, the accurate prediction and optimization of CRISPR with respect to off-target effects are essential for the successful application of CRISPR/Cas [23,24] and for future regulatory approvals for industrial applications.

The CRISPR/Cas system relies on a single guide RNA (sgRNA) sequence to direct the Cas9 enzyme to the target-specific DNA sequence. However, the accessibility of Cas9 to the target DNA site can be influenced by the chromatin architecture of, and different epigenetic modifications to, the target site [25,26]. DNA in euchromatin regions is more accessible to Cas9 than that in heterochromatin regions, where the chromatin structure is highly condensed [27,28,29]. In contrast, genes located in euchromatin regions generally exhibit higher expression levels than those located in heterochromatin regions [30,31]. Hence, we hypothesized that the chromatin state and epigenetic landscape at the target locus would be important factors influencing the accessibility and activity of the CRISPR/Cas system, thereby impacting the editing efficiency. Validation of this hypothesis was thus one of the aspects of the present study, as described below.

Here, we present a comprehensive framework for CRISPR/Cas genome editing that addresses the challenges encountered in the study of non-model organisms. In addition to describing the crucial annotation pipeline, we also elaborate on the particular obstacles that are related to the composition of the genome and their impact on the editing efficiencies of different sgRNAs. We thus present our roadmap for adopting advances in CRISPR/Cas technology to address the challenges inherent in its application to non-model organisms and to acquire a deeper understanding of the underlying genetic mechanisms in such organisms.

## 2. Results

### 2.1. Gene Structure Prediction in M. rosenbergii as an Example of a Non-Model Organism

**Figure 1:**
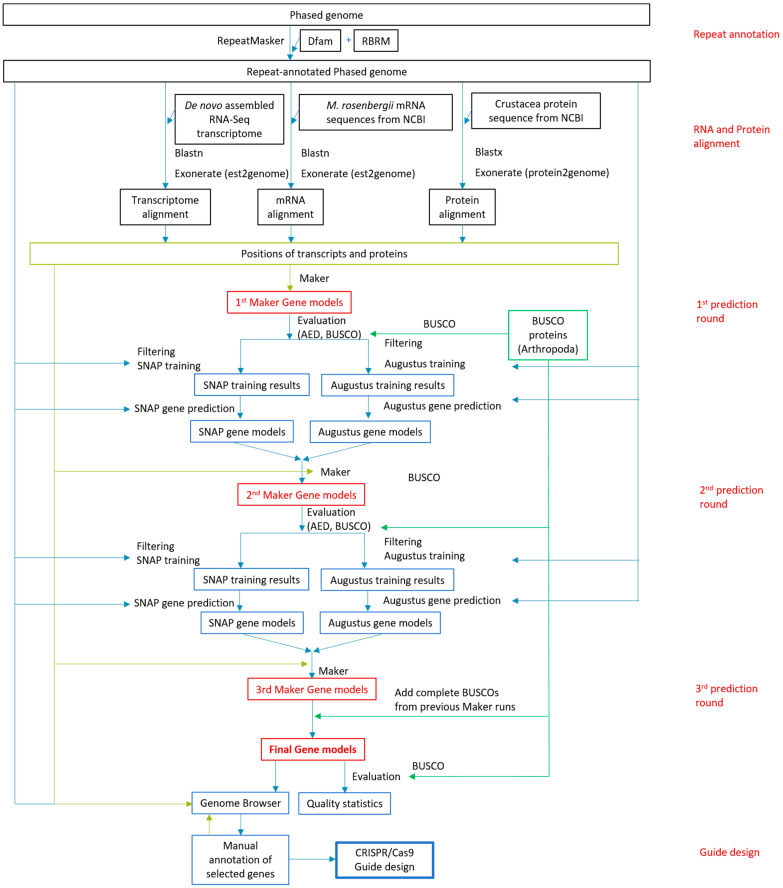
Overview of the genome annotation process proposed for CRISPR/Cas9 guide design for non-model organisms. This iterative workflow uses the example of *M. rosenbergii*, including transcript and protein alignments, along with ab initio gene predictions. The automated annotation step is followed by the manual annotation of the selected target genes by using visualization in a genome browser. BUSCO is used to evaluate the results of each iteration as well as to improve the set of final gene models.

With the aim of devising a broad scheme for CRISPR/Cas9 guide design for non-model organisms, we started the process with the high-quality annotation of the reference genome. This step was necessary because the accuracy of automated ab initio gene structure prediction software is far from perfect (see, e.g., [32]). As an example organism, we used the non-model organism, *M. rosenbergii*, whose genome poses challenges for gene structure prediction due to its high abundance of repetitive sequences, the existence of similar but non-identical sex chromosomes, and the high level of genetic heterogeneity among individuals. Therefore, to produce high-quality gene predictions for CRISPR/Cas9 guide design, we applied a two-stage process for the annotation of the reference genome. First, we used the Maker pipeline to computationally annotate the entire genome, and then we manually improved the annotation of the selected target genes through the inspection of their predicted models along with the alignment of transcripts and proteins to the same genomic region. The entire process is depicted schematically in Figure 1.

**Figure 2:**
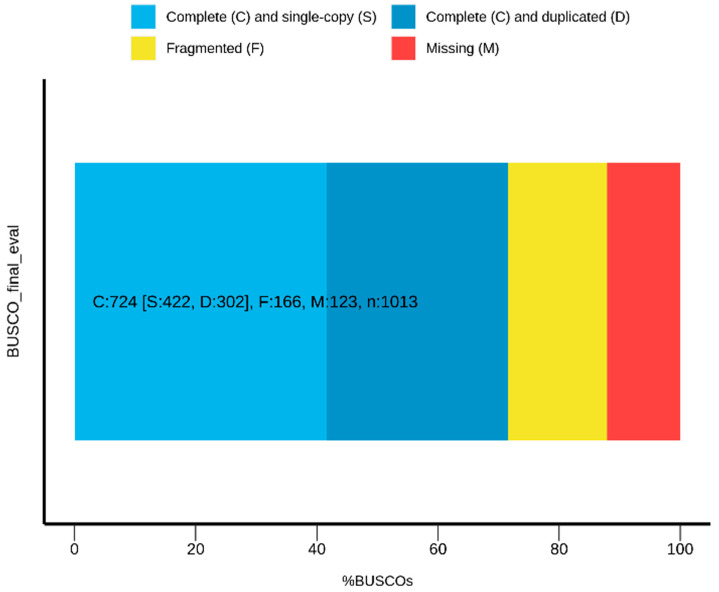
BUSCO used in the guide design for non-model organisms for the quality assessment of the final gene models. The diagram shows the proportion of BUSCO genes in the final gene models predicted in our example of the non-model *M. rosenbergii* genome. Light blue—percentage of the complete and single-copy genes in the assemblies; darker blue—percentage of the complete and duplicate genes; yellow—percentage of the fragmented genes; and red—percentage of the missing genes.

**Figure 3:**
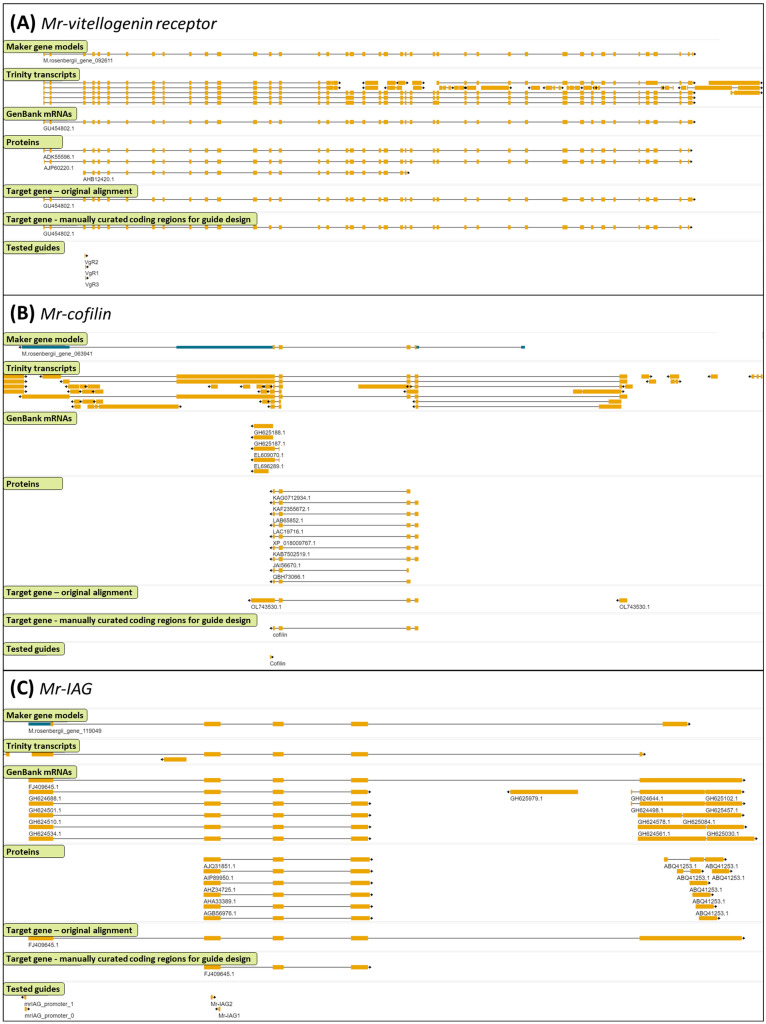
Three examples for genomic regions demonstrating various levels of agreement between the evidence alignments and the final gene model. JBrowse view of the genomic region surrounding the (**A**) *Mr-vitellogenin receptor* (GenBank GU454802.1); (**B**) *Mr-cofilin* (GenBank OL743530.1); and the (**C**) *Mr-IAG* (GenBank FJ409645.1) genes. The following tracks are displayed on the Jbrowse genome browser from top to bottom: Maker gene models—final gene predictions made by the Maker; Trinity transcripts—genome alignment of Trinity-assembled transcriptome; GenBank mRNAs—genome alignment of *M. rosenbergii* mRNA sequences from NCBI; proteins—genome alignment of the crustacean proteins from NCBI; target gene original alignment—genome alignment of the mRNA of interest; target gene manually curated coding regions for guide design—coding exon locations for guide design, based on the manual inspection of the above tracks. Tested guides represented in the bottom track. The genomic sequence of the region shown in panel (**B**), as well as the sequences and positions of the features in this region, are available in Files S1–S6.

The Maker pipeline for automated genome annotation [33,34] can run gene prediction programs, such as SNAP and Augustus, and merge their outputs with the empirical data from mRNA and protein alignment to the genome. To train the gene prediction programs using empirical data, rather than using an annotated genome from another species, we first used Maker to create gene models based solely on empirical data (first prediction round). Following filtering for high-quality models, the gene models were then used for training SNAP and Augustus. In the second prediction round, Maker executed SNAP and Augustus ab initio predictions and integrated their results with those of the empirical alignments to produce Maker gene models. It is possible to run Maker iteratively, such that the gene models produced in each iteration serve to train SNAP and Augustus in the next one. Therefore, in the second round, Maker gene models were used to train SNAP and Augustus for a third prediction round. In each Maker round, the models were evaluated using a calculated annotation edit distance (AED) score and BUSCO analysis (Table 1). In addition, we ensured that all the complete BUSCOs found in the genome in all of the three Maker rounds were included in the final gene models. The set of final gene models (Table 1, Figure 2) was uploaded to a genome browser, and additional tracks were created with the mRNA and protein alignments data. For guide design, selected genes were inspected visually on the genome browser, and, if necessary, the gene models were manually curated on the basis of all the available data. The final gene models submitted to guide design were composed solely of coding sequences (to the extent possible), with emphasis on the 5′ exons. These models were uploaded to the browser as an additional track, along with their tested guides (Figure 3).

### 2.2. Examples of Manually Curated Genes and Their Designed Guides

Figure 3 shows three example genes, representing cases of high (Figure 3A), low (Figure 3B), and intermediate (Figure 3C) agreement between the various empirical alignments and the Maker prediction. Note that, typically, genes were located on two scaffolds, which could result from the fact that a phased genome was used as a reference for the annotation. For guide design, we selected one of the scaffolds.

Figure 3A shows the genomic region of the *M. rosenbergii vitellogenin receptor* (GenBank GU454802.1). As shown in the browser, Maker predictions, transcriptome alignments, NCBI protein alignments, and alignment of the GU454802.1 mRNA, mostly agreed with one another, with evidence also being presented for some alternative splicing positions, e.g., in exons 18–19. Three crustacean proteins were found in this region, two of which were from *Macrobrachium* (ADK55596.1 of *M. rosenbergii*, which is a product of GU454802.1 mRNA, and AJP60220.1 of *M. nipponense*) and a shorter one from *Palaemon carinicauda* (AHB12420.1). The *Macrobrachium* protein alignments indicated a 3′ untranslated region (UTR) in part of the last exon. Therefore, the final manual annotation ended at the same position as the protein alignment.

Figure 3B shows *Mr-cofilin* (OL743530). As shown, the various transcript alignments were not consistent with the Maker prediction or with one another. In particular, the protein alignments presented 3 or 4 exons, with several variations in the 5′ exons (not all of them are shown in Figure 3A). Therefore, the manually annotated gene structure was based on the majority of the proteins.

Figure 3C shows *M. rosenbergii insulin-like androgenic gland specific factor* (*Mr-IAG*, GenBank FJ409645.1). The alignment of FJ409645.1 to the genome showed five exons. Other mRNAs from GenBank either lacked the last exon (5) or displayed a different exon configuration at the 3′ end, which was not included in the figure. The Trinity transcript exhibited a slightly shorter exon 1 and a very short exon 5. The Maker prediction identified a 5′ UTR in part of exon 1 and included a shorter exon 5 than that in FJ409645.1. Crustacean protein alignments revealed three exons, in the same positions as exons 2–4 of FJ409645.1. Therefore, the manually curated gene included the three exons inferred from the protein alignments.

### 2.3. Impact of Genetic Variations on the Interpretation of the Editing Results

**Figure 4:**
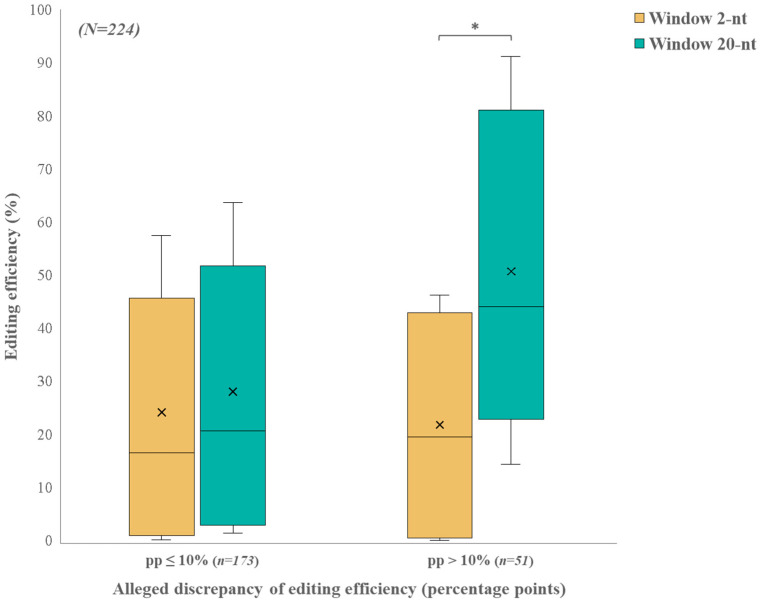
Comparison of AEE between the 2-nt and 20-nt windows for different sgRNAs. AEE distribution for 224 target sites in *M. rosenbergii* primary cell culture grouped by window size around the CRISPR/Cas9 cut-site and conditioned by their AEE difference. This comparison illustrates the distribution of the AEEs for windows 2 and 20 for two conditions: sgRNAs with ≤10 percentage points in the AEE and sgRNAs with >10 percentage points. The horizontal line within each box represents the median. The tops and bottoms of each box correspond to the 5th and 95th percentiles. The whiskers above and below each box denote the minimum and maximum values. The mean is denoted by “×”. Asterisk represents a significant difference between the distribution of the AEEs for windows 2 and 20 (Repeated measures ANOVA, *p* < 0.001).

**Figure 5:**
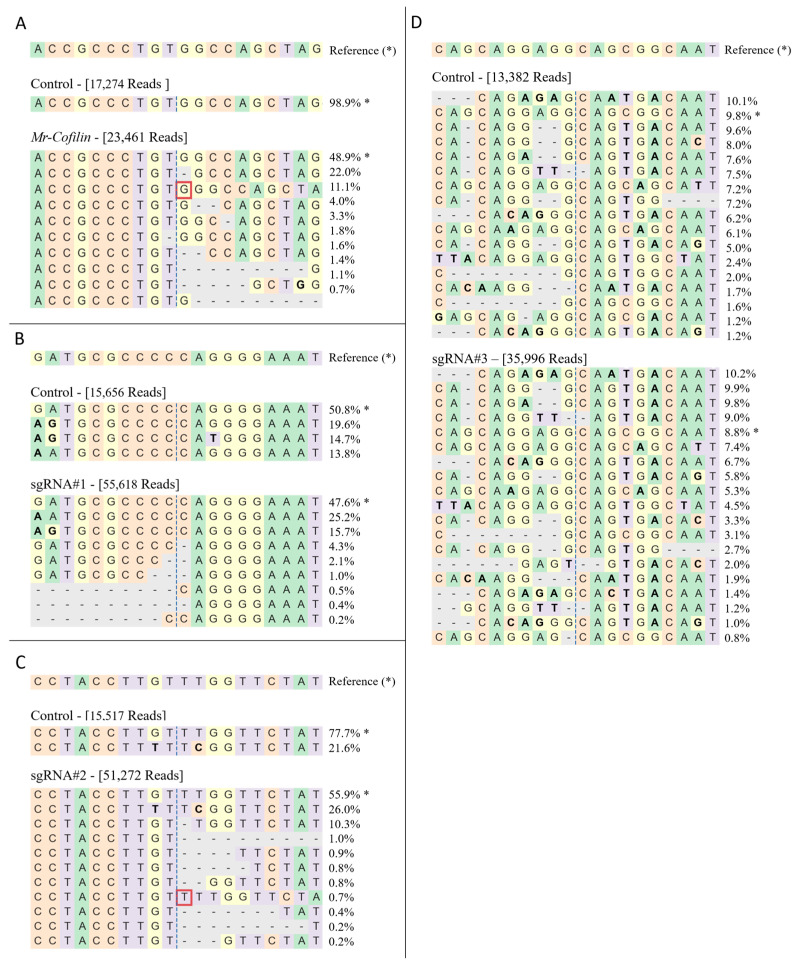
*Mr-cofilin* positive control and representative cases of genetic variations affecting the CRISPR/Cas editing analysis. CRISPResso2 outputs illustrate the distribution of identified alleles surrounding the predicted cut-site of the sgRNA. Each case represents a different source of genetic variability that causes misleading results: (**A**) sgRNA positive control, *Mr-cofilin*, (**B**) SNPs, (**C**) differences between the sex chromosomes, and (**D**) repetitive sequences. Each case is represented by a reference sequence shown in the top line and marked with an asterisk. Control and treated primary cell allele frequencies are shown below the reference sequence, with the NGS total number of reads for each. The unmodified reads are marked with an asterisk. Percentages of CRISPResso2 sequencing reads for window 20 are shown to the right of each sequence. A vertical dashed line indicates the predicted cut-site. All the sequences are color coded by nucleotide (green: A, orange: C, yellow: G, purple: T). Substitutions are given in bold letters; red rectangles indicate insertions and dashed horizontal lines indicate deletions.

To illustrate the immediate and broader effects of Cas9 activity on the genome, we conducted 16 nucleofection experiments and examined the Alleged Editing Efficiency (AEE) for 224 sgRNAs. A comparison of the AEE between the narrow and wide windows around the sgRNAs cut-site (2 nucleotides (nt) and 20 nt, respectively) showed the results were usually consistent. However, in a fraction of the cases, a large difference was revealed. A threshold of 10 percentage points between the AEE in the two windows was thus determined based on the highest discrepancy between the two analysis windows in the positive control, *Mr-cofilin*. As shown in Figure 4, in ~77% of the sgRNAs, the AEE between the two windows remained at a difference of less than ten percentage points. In contrast, the remaining sgRNAs showed a significant discrepancy in the AEE between the two analysis windows. The distribution of the AEE between window 2 and window 20 for the sgRNAs that had a difference of less than 10 percentage points was similar in the two windows. In contrast, window 20 had a significantly higher distribution than window 2 for sgRNAs with a discrepancy of more than ten percentage points (Figure 4). This unexpected observation led us to reevaluate our results. To understand the possible causes of these unexpected differences in some of the guides, we sequenced three representative loci from the matching sgRNA-free wild-type (WT) nucleofected control samples that did not include any sgRNAs (sgRNA-free), in addition to the *Mr-cofilin* controls presented in Table S1 (controls data accessible at NCBI GEO database, accession GSE281095). The sequencing results suggested three types of genetic variation that could explain the discrepancies in the AEE between the 2-nt and 20-nt windows, namely, SNPs, sex chromosome differences, and repetitive sequences (corresponding to sgRNA#1, #2, and #3, respectively; see below). For each genetic variation, the detailed next-generation sequencing (NGS) results, AEEs of sgRNA-free and sgRNA-treated samples and primers, are shown in Figure 5, Tables S2 and S3, along with *Mr-cofilin.*

The NGS results for the positive control sgRNA *Mr-cofilin* showed, as expected, real CRISPR/Cas9 editing (Figure 5A and Table S2). For the sgRNA-free sample, there were only 0.18% and 1.15% of the modified reads in windows 2 and 20, respectively. In the sgRNA-treated sample, windows 2 and 20 showed that 40.97% and 51.09%, respectively, of the total reads were modified sequences.

For sgRNA#1, which was located on an autosomal chromosome, the NGS analysis for windows 2 and 20 for the sgRNA-free sample revealed that the editing efficiency was allegedly 0.14% and 49.22%, respectively (Figure 5B and Table S2). In comparison, for the sgRNA-treated sample, the NGS analysis for windows 2 and 20 showed that 9.99% and 52.39% of the total reads, respectively, had modified sequences (Figure 5B and Table S2). The modifications in the sgRNA-free sample and some of the modifications in the sgRNA-treated sample exhibited substitutions—but no deletions—around the cut-site (Figure 5B); according to a comparison with the sgRNA-free sample, these substitutions were SNPs in the population.

For sgRNA#2, which was located on scaffolds that were identified as part of the sex chromosomes, an NGS analysis revealed that the AEE was 0.07% and 22.26%, for windows 2 and 20, respectively (Figure 5C and Table S2); in contrast, for the sgRNA-treated sample, 16.80% and 44.09% of the total reads, respectively, were modified sequences (Figure 5C and Table S2). The only false-positive modification found in the sgRNA-free sample (21.6%) matched one of the alleles in the sgRNA-treated sample (26.0%). In this specific allele, there were only substitutions (Figure 5C). Since this sgRNA was located on a sex chromosome, it is suggested that the differences between the sex chromosomes W and Z constitute the reason for the different sequences.

For sgRNA#3, NGS analysis for windows 2 and 20 for the sgRNA-free sample revealed that the editing efficiency was allegedly 10.57% and 90.17%, respectively (Figure 5D and Table S2); in contrast, in the sgRNA-treated sample, NGS analysis showed that there were modified sequences for 17.35% and 91.22% of the total reads, respectively (Figure 5D and Table S2). Different repetitive sequences could be recognized in both the sgRNA-free and sgRNA-treated samples, meaning that we could not distinguish between the actual edited sequences in the targeted location and false cases that constituted similar sequences in other locations in the genome.

### 2.4. Off-Target Editing

**Figure 6:**
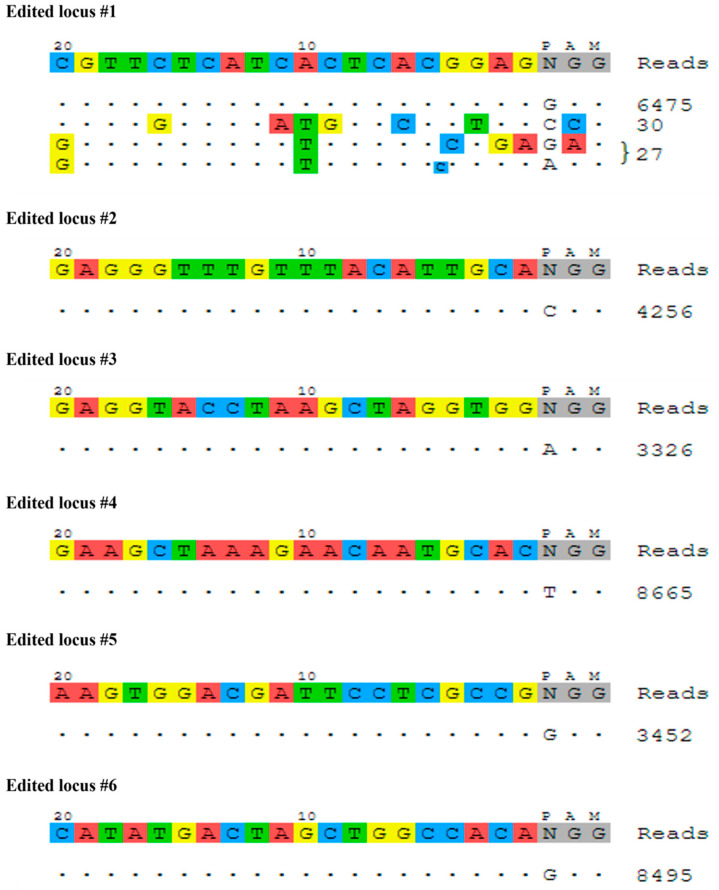
Screening off-target events in different edited loci of an *M. rosenbergii* primary embryonic cell culture. GUIDE-seq visualization showing sequences where the donor dsODN is incorporated in the genome, with reference sequence including sgRNA and PAM for each case at the top. The identified on-targets and off-targets are represented below the reference sequence. Dots indicate matching nucleotides to the sgRNA reference sequence, and in cases of mismatches to the on-target site, different nucleotides are highlighted in different colors. Reference sequences, mismatches and PAM are color coded by nucleotide (red: A, blue: C, yellow: G, green: T and gray represents the PAM). Reads on the right signify the number of reads in which the dsODN was detected.

Off-target assessment is of both basic and applied importance in model and non-model organisms. The GUIDE-seq (genome-wide unbiased identification of DSBs evaluated by sequencing) method was thus implemented for off-target identification via the insertion of double-stranded oligodeoxynucleotides (dsODN). For all the six edited loci that were examined, the donor dsODN was successfully integrated into on-target sites. For most of the targeted loci, no off-target sites were identified. The only off-target events that were discerned were those in edited locus #1, but even in this case, the number of reads for these off-target events was much lower than the number of reads for the on-target incorporations: there were 6475 reads for the on-target sequence and 57 off-target incorporations, which represented 0.0087% of the total reads (Figure 6).

### 2.5. Relation Between Gene Expression Level and Editing Efficiency

**Figure 7:**
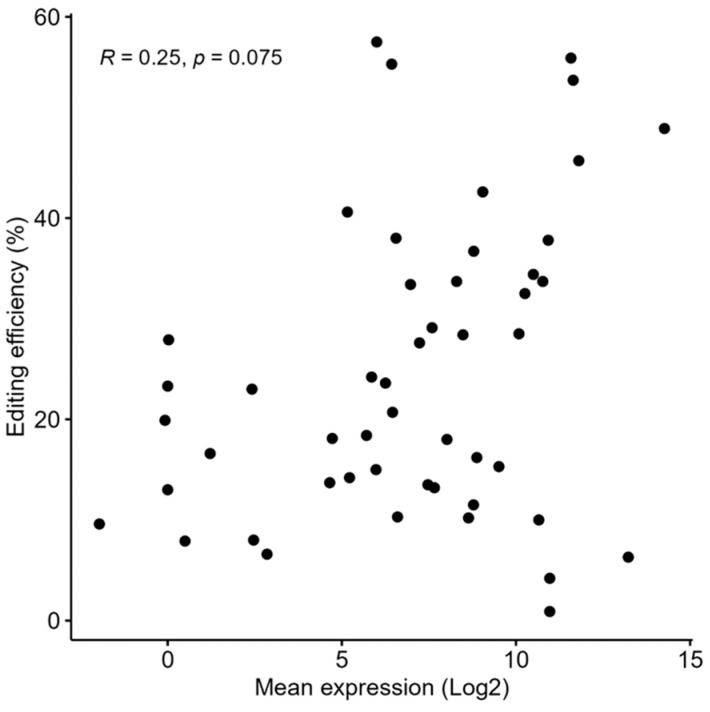
Correlation between expression levels in embryos and editing efficiency in primary embryonic cell culture. Each dot symbolizes a gene; the value in the X-axis is the log2 of the normalized gene expression level in 11-day-old embryos, and the value in the Y-axis is the gene knock-out editing efficiency in window 2.

To study the possible relationship between the expression levels and the editing efficiencies, the editing efficiencies in 50 different genes subjected to nucleofection experiments were assessed by NGS. The expression levels of these genes were retrieved from a transcriptomic library of 11-day-old embryos, which is comparable to the average day/age of the embryos that were extracted to further prepare the primary cell culture. No significant correlation was observed between the expression levels and the editing efficiencies (Spearman R = 0.254, *p*-value = 0.0747, Figure 7).

## 3. Discussion

Genome editing technologies, such as CRISPR/Cas, which have revolutionized the field of genetics, have become powerful tools for studying and manipulating the genomes of various organisms [35]. However, while the application of these technologies is advanced in model organisms, it is still limited in non-model organisms. Genome editing for non-model organisms poses challenges due to the lack of deep and ample genome sequence information and the scarcity of genetic resources, together with the high genetic diversity of non-model organisms [36,37].

In this study, we provided a generalized roadmap for establishing a CRISPR/Cas-based genome-editing platform for non-model organisms. This platform takes into consideration factors that can affect the accuracy and efficiency of the genome editing and the analysis of its results. The roadmap rests on three pillars: using genome annotation, particularly within coding regions (which is crucial for accurate CRISPR/Cas9 guide design); leveraging published *M. rosenbergii* genome assemblies (NCBI RefSeq assembly: GCF_040412425.1, Submitted GenBank assembly: GCA_040167855.1; GCA_039081455.1); and using automated gene prediction that incorporates all available information, followed by manual curation. This information may include transcriptome assemblies from an array of public and in-house RNA-Seq datasets and GenBank mRNA and protein sequences [38,39,40]. While a BUSCO analysis and manual inspection through a genome browser indicated overall good quality, some uncertainties remained in the gene structure predictions, necessitating further refinement. Therefore, to ensure guide design accuracy, we meticulously reviewed and manually improved the annotation of the target genes of interest on the genome browser by using all available evidence. The suggested scheme for gene prediction and manual refinement, which enabled us to design and test the guides, may be a valuable tool for editing the genomes of non-model organisms.

For most of the examined sgRNAs from the prawn embryonic cell culture, the AEE was consistent between the narrow window (2 nt) and the wider window (20 nt) around the cut-site. Nevertheless, for some of the examined sgRNAs, there was a significant discrepancy in the AEE between the narrow and wide windows, with the AEE in the 20-nt window being significantly higher. These findings indicate the importance of using both narrow and wide regions around the Cas9 cut-site to assess the immediate effects of Cas9 activity and to evaluate sgRNA editing efficiency [41], since discrepancies between the windows might indicate false editing results, which may be misleading when evaluating the real editing efficiency of a sgRNA.

The unexpected discrepancies in the editing efficiencies of the different sgRNAs found in this study may be attributed to genetic variations in the target regions, namely, to the abundance of SNPs, to the differences between the sex chromosomes, and to the occurrence of repetitive sequences. Thus, of the three representative examples of discrepancies in AEEs, the first related is to SNPs, which are widely distributed throughout the genomes of most organisms [42]. This autosomal chromosome example showed that the occurrence of SNPs seemingly led to high AEE in the 20-nt window, while it was low in the 2-nt window. Similarly, the second example taken from a sex chromosome showed that the differences between the W and Z chromosomes seemingly led to relatively high AEE in the 20-nt window compared to very low efficiency in the 2-nt window. The third example showed that repetitive sequences may contribute to false analyses in genome editing, making it difficult to distinguish between actual targeted sequences and similar sequences found elsewhere in the genome. We note that repetitive sequences are ubiquitous features in many genomes [18,43], with some non-model arthropod species harboring remarkably high levels compared to the model organisms [44]. The above three representative examples underscore the importance of taking the genetic background of the target regions into consideration when interpreting CRISPR/Cas editing results in any organism. The examples presented here demonstrate the importance of deep sequencing the WT amplicon in such cases, as pre-existing genetic variations can significantly impact the observed outcomes, especially when analyzing a wide genomic window around the target site.

The rates of off-target events may affect the accuracy and applicability of genomic editing [45,46] and may also give rise to regulatory concerns. In this study, off-target profiling for *M. rosenbergii* indicated that the CRISPR/Cas gene editing was precise, with successful integration of the donor dsODN at the intended on-target sites for all six of the edited loci studied. Importantly, the off-target effects were minimal across most of the targeted loci. The total number of off-targeted events in the present study was much lower than that for the on-target integrations. Only three off-target sites were identified, demonstrating ~0.0087% of the total reads. In this study, the off-target rates are considered very low compared to another study that exhibited as high as 150 sites of off-targets for one of multiple investigated cases [46]. To the best of our knowledge, our work presents the first use of GUIDE-seq for detecting off-targets in arthropods. Previous GUIDE-seq studies have primarily focused on human cell lines and a limited number of other mammalian cells, such as mouse primary cells [46,47,48]. More commonly used traditional methods for detecting off-targets have relied on predicting and examining potential off-target sites by sequencing [49,50]. While informative, these methods often have sensitivity and genome-wide coverage limitations. Our application of GUIDE-seq in *M. rosenbergii* represents a significant advancement in the ability to comprehensively assess off-target events in arthropods, potentially improving the safety and efficacy of CRISPR-based gene editing in non-model organisms. GUIDE-seq is considered one of the most sensitive methods for detecting off-target effects in human cells [50]. Combining this method with our comprehensive roadmap that leads to precise sgRNA design might have resulted in a specific design that enabled the achievement of relatively low off-target rates. Importantly, the prevention of off-target effects is a critical aspect of ensuring safety in CRISPR-based modifications [6,51,52] and should be an important consideration in any future manipulation.

Finally, it was hypothesized that the chromatin state and epigenetic landscape at the target locus would influence the accessibility and activity of the CRISPR/Cas system, thereby impacting the editing efficiency of the sgRNAs [26,29]. Thus, it was posited that the target genes accompanied by epigenetic and chromatin architecture favoring high transcription levels [53] would be correlated with high editing success. Nonetheless, the results presented here for the study of 50 genes found no significant correlation between expression levels and editing efficiency of the CRISPR/Cas system, thereby contradicting the above hypothesis. Interestingly, although the expression levels of the studied genes did not correlate with editing efficiency, the scatter plot in Figure 7 might suggest an ostensible bifurcation behavior, implying that some data points are positively correlated while others are negatively correlated. This divergence might suggest that other factors, such as the specific chromatin structure, epigenetic modifications, and the target sequence composition [27], may affect the efficiency of CRISPR-mediated genome editing. Thus, further research is needed to elucidate the complex interplay between the chromatin structure, epigenetic modifications, expression levels, and the accessibility and activity of the CRISPR/Cas system. Exploring these relationships in greater detail could lead to improved strategies for targeted genome editing, ultimately enhancing the precision and efficiency of CRISPR-based applications.

In summary, overcoming the above-presented challenges in the non-model organism *M. rosenbergii* can provide valuable insights for CRISPR applications in other crustaceans and other non-model organisms towards establishing a robust CRISPR/Cas genome editing platform. Developing tools for genome editing in non-model organisms, including crustaceans, has the potential to improve important traits for aquaculture [54,55], specifically, in aquaculture animals such as *M. rosenbergii,* for which there has been an increase in genomic sequencing efforts [39,56]. By identifying and characterizing the genetic variations in non-model species, we can better understand the genes and pathways controlling important traits, such as growth, disease resistance, and environmental adaptability [6]. This will enhance our understanding of the genetic landscape, improving the efficiency and precision of genome editing, and enabling the application of transformative genomic approaches to non-model species, in general, and aquaculture species, in particular. The present research presents a tentative generalized road map for establishing a CRISPR/Cas-based genome-editing platform for non-model organisms and provides valuable insights into factors that can influence the success of CRISPR/Cas genome editing, thereby aiding researchers and other end users to overcome the challenges encountered in studying and manipulating non-model organisms.

## 4. Materials and Methods

### 4.1. Assembly of CRISPR/Cas Guide Design Scheme for the Non-Model Organism M. rosenbergii

As a representative non-model organism, we used *M. rosenbergii,* whose assembled genome [39] was de novo annotated using Maker v2.31.9 [33,34]. Maker is a genome annotation pipeline performing empirical and ab initio gene prediction using and integrating existing software tools. As the reference genome, we used a phased genome assembly consisting of scaffolds, each representing a single, phased haplotype [39]. Thus, each genomic locus might be represented by more than one scaffold. Below is a detailed description of the annotation process up to the CRISPR/Cas9 guide design. A schematic representation of the entire suggested workflow for *M. rosenbergii*, which is applicable to any non-model organism, is provided in Figure 1. The Maker annotation code was written according to the following tutorials: “Genome Annotation using MAKER” and “Tutorial of how to run Maker2 gene annotation pipeline” [57,58].

### 4.2. Preliminary Steps

RepeatMasker v4.1.5 [59] was used to identify low-complexity simple repeats, e.g., mononucleotide runs and microsatellites, and complex interspersed repeats, e.g., transposons and retrotransposons. Crustacean repeat elements from two databases, RepBase RepeatMasker edition (final version 26 October 2018) and Dfam v3.2 (2 July 2020), were used for the analysis. To enhance the gene prediction with empirical data and to perform the initial training of the ab initio prediction programs, we aligned three sets of mRNA and protein sequences to the repeat-annotated genome: (1) The Trinity de novo [60]-assembled transcriptome of *M. rosenbergii* (1,513,631 contigs) produced by RNA-Seq reads from previous experiments performed in our laboratory and elsewhere [38,40]; (2) a total of 9112 *M. rosenbergii* mRNA sequences from the NCBI Nucleotide database, retrieved using the search term “(“*Macrobrachium rosenbergii*” [Organism] OR *Macrobrachium rosenbergii* [All Fields]) AND biomol_mrna[PROP]”; and (3) a total of 1,291,174 crustacean protein sequences from the NCBI Protein database, retrieved using the search term “Crustacea.” The mRNA and protein sequences were aligned to the repeat-masked genome using blastn and blastx, respectively. The alignments were polished using Exonerate [61] est2genome and protein2genomes models, respectively. Exonerate realigns each sequence identified by BLAST around splice sites and forces the alignments to occur in order.

### 4.3. First Prediction Round

Using mRNA and protein positions, along with repeat annotations, Maker generated initial gene models and calculated an AED quality measure per model. This yielded 496,476 gene models with a median length of 437 bp, out of which 96% had an AED < 0.5. BUSCO analysis [62] using the BUSCO arthropoda_odb10 database showed 64.9% complete BUSCOs (about half single copy and half duplicated) and 17.1% fragmented BUSCOs (Table 1). These gene models were used for training the ab initio gene prediction software SNAP [63] and Augustus v3.5.0 [64]. For SNAP v2013-11-29 training, we used gene models having AED ≤ 0.25 and a length of 50 or more amino acids, plus 1000 bp from both sides of the gene model for training the intergenic regions. The final hidden Markov models (HMMs) from SNAP training were assembled and used as inputs for the SNAP second prediction round.

Augustus training accepts a smaller set of gene models, which are non-redundant, non-overlapping, and as accurate as possible and are represented by only one splice variant. Therefore, using the AGAT package [59,65] and in-house scripts, the following filtering criteria were applied: (a) AED value = 0; (b) the selection of isoforms with the longest coding sequence (CDS) per gene; (c) the use of complete protein-coding genes, having both start and stop codons; (d) the filtering of the genes to include only those with UTRs flanking both the 5′ and 3′ ends of the coding sequence; (e) the selection of genes that were at least 500 bp apart from their neighbors (to facilitate proper training of intergenic regions); and (f) non-redundancy, which was ensured by performing a reciprocal blastp of the translated gene models and keeping a representative gene per group of similar genes.

The top 1000 genes having the largest sum of lengths of 3′ and 5′ UTRs were selected and randomly divided into training (900 genes) and test (100 genes) sets. After initial training and optimization of the meta-parameters, Augustus was used to predict the gene models in the test set to assess the prediction accuracy. In the test set, 19% of the genes and 49.3% of the exons were predicted accurately. The final parameters were used as inputs for the second prediction round of Augustus.

### 4.4. Second Prediction Round

In the second prediction round, SNAP and Augustus were executed to perform ab initio gene prediction using the training results from the previous round. As shown in Table 1, the prediction resulted in a reduction in the number of gene models (209,497) and a longer median length (1106 bp) than in the first round. However, the percentage of genes with AED < 0.5 decreased to 70%. The number of complete BUSCOs and the fraction of single copy BUSCOs improved slightly (68% and 35.7%, respectively, Table 1). The positions of the SNAP- and Augustus-generated gene models, along with the positions of the aligned mRNAs and proteins (from the preliminary steps), were subsequently processed by Maker to produce improved gene models. The Maker gene models were filtered and then submitted for the training of SNAP and Augustus, as above. In the Augustus test set, 33% of the genes and 75% of the exons were predicted accurately.

### 4.5. Third Prediction Round

Ab initio gene prediction was once again performed by SNAP and Augustus, this time using the training results from the second prediction round. The positions of the generated gene models, along with the empirical protein and mRNA alignments, were then processed by Maker to produce third-round gene models. As shown in Table 1, the third prediction round slightly improved the results compared to the second round, with fewer and longer genes, and a higher percentage of genes with AED < 0.5. We found that some gene models from the first and second rounds that completely matched the BUSCO proteins were missing in the third-round gene set. Since these were presumably reliable gene models, we added them to the final gene model set. This added 41 complete BUSCOs and 53 gene models to the final set and resulted in improved BUSCO statistics for the final gene set compared to each of the three prediction rounds (71.5% complete BUSCOs, 41.7% single-copy, and less fragmented and missing BUSCOs compared to the first and second rounds; see Table 1 and Figure 2).

### 4.6. Further Manual Annotation of Selected Target Genes and Guide Design

A set of selected target transcripts (from the “Embryo transcriptome” [38] and from NCBI) for which we wanted to design CRISPR/Cas9 Guides were aligned to the genome by using minimap2 v2.13. All the data that had been collected—including the final Maker predictions, the empirical alignments of the crustacean proteins, the *M. rosenbergii* mRNAs, the Trinity-assembled transcriptome, and the alignment of the selected target genes—were uploaded as individual tracks to a local installation of the JBrowse Genome Browser [66]. By manual inspection of all the alignments in the genomic regions of the target genes, we defined the final gene models to be submitted to guide design. The final gene models were composed only of coding sequences (to the extent possible), with emphasis on the 5′ exons. CRISPR/Cas9 Guide design was performed using the IDT CRISPR/Cas guide RNA design tool [67]. The sgRNAs and primers that were used for Figure 3 are available in Table S4.

### 4.7. Animal Maintenance

Eight breeding groups of *M. rosenbergii* were grown in 500 L tanks at 27 ± 2 °C, with a light–dark regime of 14:10 and constant aeration; the tanks were held in a dedicated facility at Ben-Gurion University of the Negev (BGU). Each group comprised one *M. rosenbergii* male and up to seven females per tank. All the groups were fed on a daily basis with dry and frozen pelleted food, and checked to track gravid females. Gravid females were transferred singly to 100 L aquaria with internal filtration and aeration until their embryos were collected and used for cell extraction.

### 4.8. Cell Extraction

Prior to the isolation of the embryonic cells, *M. rosenbergii* egg-bearing females were disinfected in a methylene blue water bath (up to 10 drops of methylene blue to 10 L water) at least one day prior to cell extraction. Eggs were collected from a single female abdomen for each experiment and further disinfected by washing for 10 min in a rotator in crustacean physiological saline (CPS) [68], antibiotics (PEN/STREP, Biological Industries, Beit HaEmek, Israel), and 0.5 µg/mL of the antifungal preparation, Voriconazole (Sigma, St. Louis, MO, USA). Thereafter, the eggs were strained, washed, transferred to sterile Eppendorf tubes, and homogenized, and collected, as described in Molcho et al. [11]. The cells were subjected to nucleofection 24 h post extraction.

### 4.9. Cell Nucleofection

A primary cell culture was established from the cells extracted from 10- to 14-day-old embryos, as described in Molcho et al. [11], and ribonucleoproteins (RNPs) were inserted by nucleofection. After nucleofection, the cells were transferred into a 96-well plate and incubated for recovery at 28 °C and a CO_2_ concentration of 5%. After 72 h, the cells were collected, and DNA was extracted [11]. DNA was then used as a template for amplification via a polymerase chain reaction (PCR) with specific primers flanking the sgRNAs. The PCR products were cleaned using EPPiC Fast (A&A Biotechnology, Gdansk, Poland) and subjected to Sanger sequencing at the BGU Sequencing Unit. A negative control and the *Mr-cofilin* positive control were also subjected to Sanger sequencing to monitor the editing success before proceeding to NGS. After validating the editing success, PCR products using the genomic DNA as a template were sent to the Technion–Israel Institute of Technology (Haifa, Israel) for NGS (Miseq Run V2; 2 × 150 bp, assuming 4 M reads per ends per run).

### 4.10. Cell Nucleofection Experimental Design

The 16 cell nucleofection experiments incorporated 224 sgRNA along with positive and negative controls for each experiment. The negative control comprised nucleofected WT cells without sgRNA, and the positive control comprised nucleofected WT cells with *Mr-Cofilin* sgRNA (Table S1 and accession series: GSE281095). Both the negative and positive control were sequenced for *Mr-cofilin*. Out of the 224 sgRNAs, three sgRNAs were chosen as representative cases of the different genetic variations and each WT amplicon was sequenced.

### 4.11. Next-Generation Sequencing Analysis

The NGS results were analyzed by CRISPResso2 [69]. The examination was conducted in a narrow analysis window of 2 nucleotides around the Cas9 nuclease cleavage site and a wider window of 20 nucleotides around the cleavage site. These two windows were chosen so as to provide a comprehensive view of the editing process. AEE was calculated by the total number of reads that were classified as modified (have an insertion/deletion/substitution in the quantification window) out of the total aligned reads (modified and non-modified) to the region of interest. In experiments in which editing was performed, the AEEs represented both real and false editing, and in cases where no editing was performed, these represented only false editing efficiency. A threshold value of 10 percentage points was chosen for sorting the samples in two groups (percentage points ≤ 10/percentage points > 10) in addition to the window size conditions (window 2 nt/window 20 nt). The threshold was set based on the largest discrepancy in the percentage points in the control of *Mr-cofilin*.

### 4.12. Cell Nucleofection with dsODN

Primary cell culture nucleofection was performed, as described in Molcho et al. [11], along with dsODN that was formed by annealing two modified oligonucleotides [46]. Nucleofection was performed using Lonza 100-µL Nucleocuvette Vessels and the 4D-Nucleofector™ Core and X Unit (AAF-1003B, AAF-1003X; Lonza, Basel, Switzerland). The complex was obtained by incubating a mixture of 6 µL of Cas9 [62 µM] (IDT), 4 µL of sgRNA [100 µM] (IDT), 8 µL of dsODN [50 µM] (IDT), and 25 µL of P3 nucleofection buffer (Lonza P3 Primary Cell 4D-Nucleofector^TM^ X Kit S) at 37 °C for 25 min. After incubation of the RNP-dsODN mix, 4 × 10^6^ cells diluted in 57 µL of P3 nucleofection buffer were added to the mix to bring the total reaction volume to 100 µL. The cells were electroporated using the built-in program CL-137 on the 4D-Nucleofector (Lonza). After nucleofection, the cells were reinvigorated with 650 µL of Opti-MEM in a 48-well plate and incubated for recovery at 28 °C and a CO_2_ concentration of 5%. After 72 h, the cells were collected, and genomic DNA was extracted using DNeasy Blood & Tissue Kit (QIAGEN, Hilden, Germany). Sanger sequencing was conducted to validate the dsODN integration into the on-target site. The Sanger sequencing results were analyzed using the TIDE web tool v3.3.0 [70]. After confirming the dsODN integration, the extracted genomic DNA was sent to Hylabs (Rehovot, Israel) for GUIDE-seq library preparation, as described in Tsai et al. [46]. The GUIDE-seq library was analyzed, as described by Tsai et al. [71], by the Bioinformatics Core Facility at BGU.

### 4.13. Statistical Analysis of Editing Efficiency

Statistical analyses were performed using Statistica v14.0 software (StatSoft, Ltd., Tulsa, OK, USA) to evaluate the differences in the AEE across the different experimental conditions (window size) and groups (percentage points discrepancy). Prior to the analysis, AEE percentage data were transformed using arcsine transformation to meet the assumptions of the parametric tests. The homogeneity of variance was assessed using Levene’s test. A repeated measures analysis of variance (ANOVA) was then conducted to compare the AEEs.

### 4.14. Assessing the Correlation Between Editing Efficiency and Gene Expression

RNA-Seq RSEM counts from six (three male and three female) 11-day-old embryos [38] were normalized and transformed using a regularized logarithm (rlog) in DESeq2. The expression level for each transcript was represented by the mean of the six embryos. A total of 50 genes and their guide and amplicon sequences from the editing experiments were searched against the embryo transcriptome using blastn to associate one transcript per edited gene. AEEs were calculated for a window size of 2 nt around the Cas9 nuclease cleavage site, as described above. The Shapiro–Wilk test for normality showed that the editing efficiencies did not follow a normal distribution; therefore, the correlation between editing efficiencies and the expression levels was calculated using Spearman’s rank correlation.

### 4.15. Data Availability

To illustrate the annotation and editing process, we provided Supplementary Files containing sequences and positional data related to the *Mr-cofilin* gene region displayed in Figure 3B. The files are File S1: genomic sequence of the region surrounding the *Mr-cofilin* gene, shown in Figure 3B; File S2: sequences of the Trinity-assembled transcripts aligned to this region; File S3: the GenBank accession numbers for the mRNA and protein sequences aligned to this region; File S4: the mRNA sequence of the Maker-predicted gene in this region; File S5: the protein sequence of the Maker-predicted gene in the region; and File S6: the GFF file indicating the locations of all the features in Files S2–S5, and the manually annotated gene and sgRNA within the genomic region presented in File S1. The coordinates started from the beginning of this genomic region. Moreover, the complete code for the genome annotation pipeline of this study is available at https://github.com/bioinfo-core-BGU/Genome_annotation_with_Maker (accessed on 14 November 2024).

The raw and processed data for the positive and negative controls of the 16 experiments are available in NCBI’s Gene Expression Omnibus (GEO) and accessible through GEO Series accession record GSE281095.

## Figures and Tables

**Table 1:** Evaluation of the gene models after each prediction step in the de novo genome annotation process.

	Prediction Round 1	Prediction Round 2	Prediction Round 3	Final
Total (bp)	783,943,339	704,618,362	774,562,834	775,570,631
Count (# of Genes)	496,476	209,497	179,805	179,858
Mean (bp)	1579	3363	4307	4312
Median (bp)	437	1106	1788	1788
Min (bp)	2	86	86	86
Max (bp)	120,143	379,917	291,991	291,991
% of Genes with AED < 0.5	96%	70%	72%	72%
Complete BUSCOs	64.9%	68.0%	67.4%	71.5%
Single-Copy	32.3%	35.7%	38.7%	41.7%
Duplicated	32.6%	32.3%	28.7%	29.8%
Fragmented	17.1%	18.3%	18.7%	16.4%
Missing	18.0%	13.7%	13.9%	12.1%

## Data Availability

The original contributions presented in the study are included in the article and Supplementary Materials, further inquiries can be directed to the corresponding author.

## Supplementary Materials

The following supporting information can be downloaded at https://www.mdpi.com/article/10.3390/ijms252312530/s1.
